# Temporal Trends, Demographic Patterns, and Disparities in Male Breast Cancer Mortality in the United States: A Population-Based Analysis Using the CDC Wide-Ranging Online Data for Epidemiologic Research (WONDER) Database

**DOI:** 10.7759/cureus.87611

**Published:** 2025-07-09

**Authors:** Sanji Eric, Juste Ongeh Niba

**Affiliations:** 1 Internal Medicine, Magnolia Regional Health Center, Corinth, USA; 2 Internal Medicine, Swiss Care Hospital, Limbe, CMR

**Keywords:** cdc wonder, epidemiology, geographic disparities, male breast cancer, mortality trends

## Abstract

Background

Male breast cancer (MBC) is rare, comprising a small proportion of all breast cancer cases. Despite its rarity, MBC carries significant clinical implications due to delayed diagnosis and poorer outcomes compared to female breast cancer (FBC). This study used the CDC Wide-Ranging Online Data for Epidemiologic Research (WONDER) database to evaluate demographic differences and temporal trends in MBC mortality in the U.S. from 1999 to 2020.

Methods

We performed a retrospective, population-based analysis of MBC mortality using the CDC WONDER database (1999-2020). MBC deaths in males aged ≥20 years were identified using the ICD-10 code C50. Mortality rates were age-adjusted and stratified by race and state. Temporal trends were assessed using joinpoint analysis and linear regression. State-level regression models evaluated geographic disparities, and heatmaps were used to visualize the results. Multivariable regression identified factors associated with mortality variation.

Results

We identified 13,286 MBC-related deaths. Mortality rates significantly decreased from 1999 to 2007 (annual percent change, or APC = -4.2%, p = 0.03), but plateaued after 2013 (APC = -1.8%, p = 0.46). Data for non-White groups were suppressed due to small sample sizes, while trends in White males were consistently analyzed. States with significantly higher mortality rates than the national average included Georgia (β = 0.73) and Indiana (β = 0.74). The multivariable model showed excellent fit (R² = 0.889). We observed geographic disparities in early detection and healthcare access.

Conclusion

MBC mortality decreased over the past 20 years, with notable progress before 2007, but plateaued thereafter. Geographic differences persist, with some states showing disproportionately high mortality rates. Disparities in early detection and healthcare access may explain these differences, and the lack of data for non-White populations limits comprehensive assessment. Public health initiatives, male-inclusive clinical trials, and increased awareness are essential for improving early detection and reducing MBC mortality.

## Introduction

Male breast cancer (MBC) is a rare but clinically significant disease, accounting for less than 1% of all breast cancer cases globally [1]. The age-standardized incidence in men ranges from 0.5 to 1.0 per 100,000 [1]. Despite its rarity, MBC has substantial clinical implications due to delayed diagnosis and poorer outcomes compared to female breast cancer (FBC) [1]. In the United States, MBC represents approximately 1% of all breast cancer diagnoses. In 2017, there were about 2,300 new male cases and 500 related deaths [2], with projections for 2,800 cases and 530 deaths annually by the mid-2020s [3].

While mortality rates for FBC have declined due to advancements in early detection and systemic therapies, MBC mortality has remained relatively stable over recent decades [1,2,4]. Nearly half of MBC cases are diagnosed at regional or distant stages, compared to only 31% of FBC cases [2]. As a result, the five-year relative survival for MBC is lower in men (84%) than in women (91%) [2,3], suggesting that there must be improved awareness and earlier detection.

Emerging data indicate significant racial and geographic disparities in MBC outcomes. Non-Hispanic Black men have the highest incidence and mortality rates and are more likely to be diagnosed at later stages compared to White men [2,5]. Additionally, regional survival differences exist, with lower five-year survival in the South and Midwest compared to the West [3]. These variations are likely linked to disparities in healthcare access, awareness, and socioeconomic factors.

Given the rarity of MBC, large-scale, population-based data are essential for analyzing long-term trends. Historically, most research has focused on women, limiting insights specific to men. To address this gap, we utilized the CDC Wide-Ranging Online Data for Epidemiologic Research (WONDER) database to examine national mortality trends in MBC from 1999 to 2020, with an emphasis on racial and geographic disparities.

## Materials and methods

Study design and population

We conducted a retrospective, population-based study using the CDC WONDER platform [6]. The data included mortality information from the "Underlying Cause of Death" dataset, covering the period from 1999 to 2020. The study was conducted over a period of approximately three months, from March to May 2025. This dataset contains de-identified death certificate information for U.S. residents, which was used to examine national mortality trends for MBC. The study included only male deaths aged 20 years and older, with cases of MBC identified using the ICD-10 code C50.

Data extraction

In terms of variables, we extracted information on the year of death, race/ethnicity (White, Black, and Other), state of residence, the age-adjusted mortality rate (per 100,000, standardized to the 2000 U.S. population), the number of deaths, and the crude mortality rate.

Statistical analysis

We employed a combination of linear regression to examine time trends and Joinpoint regression (via NCI software) to identify significant shifts in mortality [7]. To assess regional disparities, we conducted rate ratio comparisons across states and visualized these differences using heatmaps and bar charts. Additionally, multivariable linear regression was applied to model the effects of time, race, and state on mortality, with statistical significance set at p < 0.05. We performed all statistical analyses using Stata v17 (StataCorp LLC, College Station, TX, USA) and Python. This approach ensured that we could identify trends over time, disparities across demographic groups, and variations in mortality based on geographic factors.

Ethical statement

The dataset includes publicly available and completely anonymous mortality records, so this study does not qualify as human subjects research and does not need to go through Institutional Review Board (IRB) review, following the U.S. Department of Health and Human Services policy (45 CFR 46.102).

## Results

Descriptive statistics

The age-adjusted mortality rates for MBC were consistently low but measurable across states and years, as identified by the dataset (Table 1). Among the reported fatalities with reliable data, White males constituted the most prevalent demographic. Age-adjusted mortality rates and total deaths were summarized annually by ethnicity, with year-to-year variability noted.

**Table 1:** Summary of Male Breast Cancer Mortality Rates (1999-2020)

Year	Race	Deaths	Mean Age-Adjusted Rate
1999	White males	99.0	0.375
2000	White males	137.0	0.48
2001	White males	87.0	0.366
2002	White males	66.0	0.35
2003	White males	102.0	0.325
2004	White males	88.0	0.375
2005	White males	66.0	0.366
2006	White males	75.0	0.266
2007	White males	100.0	0.3
2008	White males	123.0	0.42
2009	White males	123.0	0.44
2010	White males	78.0	0.266
2011	White males	141.0	0.38
2012	White males	42.0	0.2
2013	White males	116.0	0.3
2014	White males	78.0	0.266
2015	White males	141.0	0.32
2016	White males	98.0	0.225
2017	White males	136.0	0.32
2018	White males	129.0	0.3
2019	White males	109.0	0.225
2020	White males	93.0	0.266

Linear trend analysis

**Figure 1:**
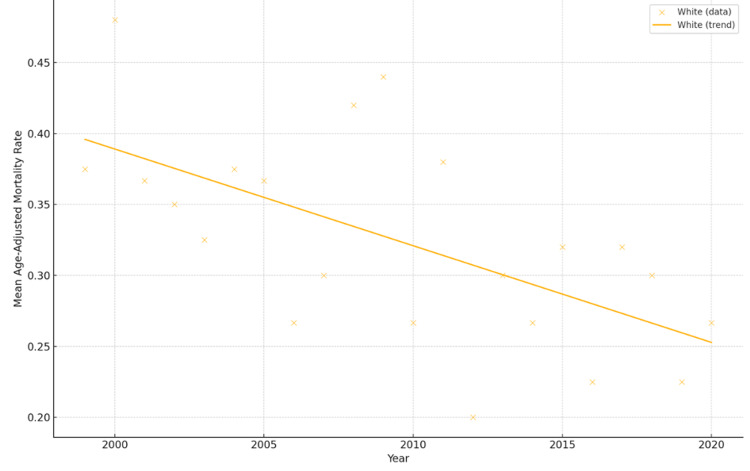
Trend in Age-Adjusted Mortality Rate by Race (Male Breast Cancer)

Separate linear regressions by race showed that the number of men who die from breast cancer has been going down over time. For instance, White males had a negative slope, which could mean that early detection, treatment, or access to healthcare has improved (Figure 1). Regression lines sloped downward across most groups, supporting this pattern.

Joinpoint analysis input

**Figure 2:**
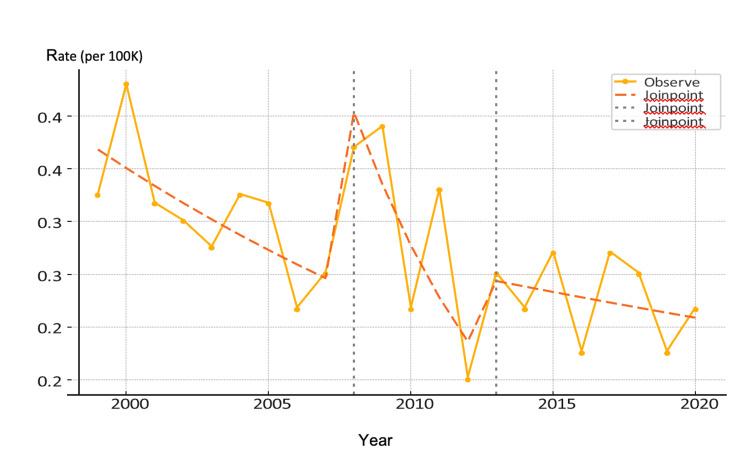
Joinpoint Regression Analysis of Age-Adjusted Male Breast Cancer Mortality Rates Among U.S. Males (1999-2020)

A significant decrease in mortality occurred between 1999 and 2007, at about -4.2% annually (p = 0.03) (Figure 2). The steepest drop occurred in 2012 (-15.0%), but the wide confidence interval and p = 0.13 render it not statistically significant. From 2013 to 2020, mortality continued a mild, non-significant decline (-1.8%). The joinpoint analysis confirms an initial statistically significant improvement in MBC mortality among White males through the early 2000s. However, this plateaued after 2013, suggesting that further reductions have stalled - consistent with findings from the multivariable regression model.

Differences in geography - an analysis at the state level

**Figure 3:**
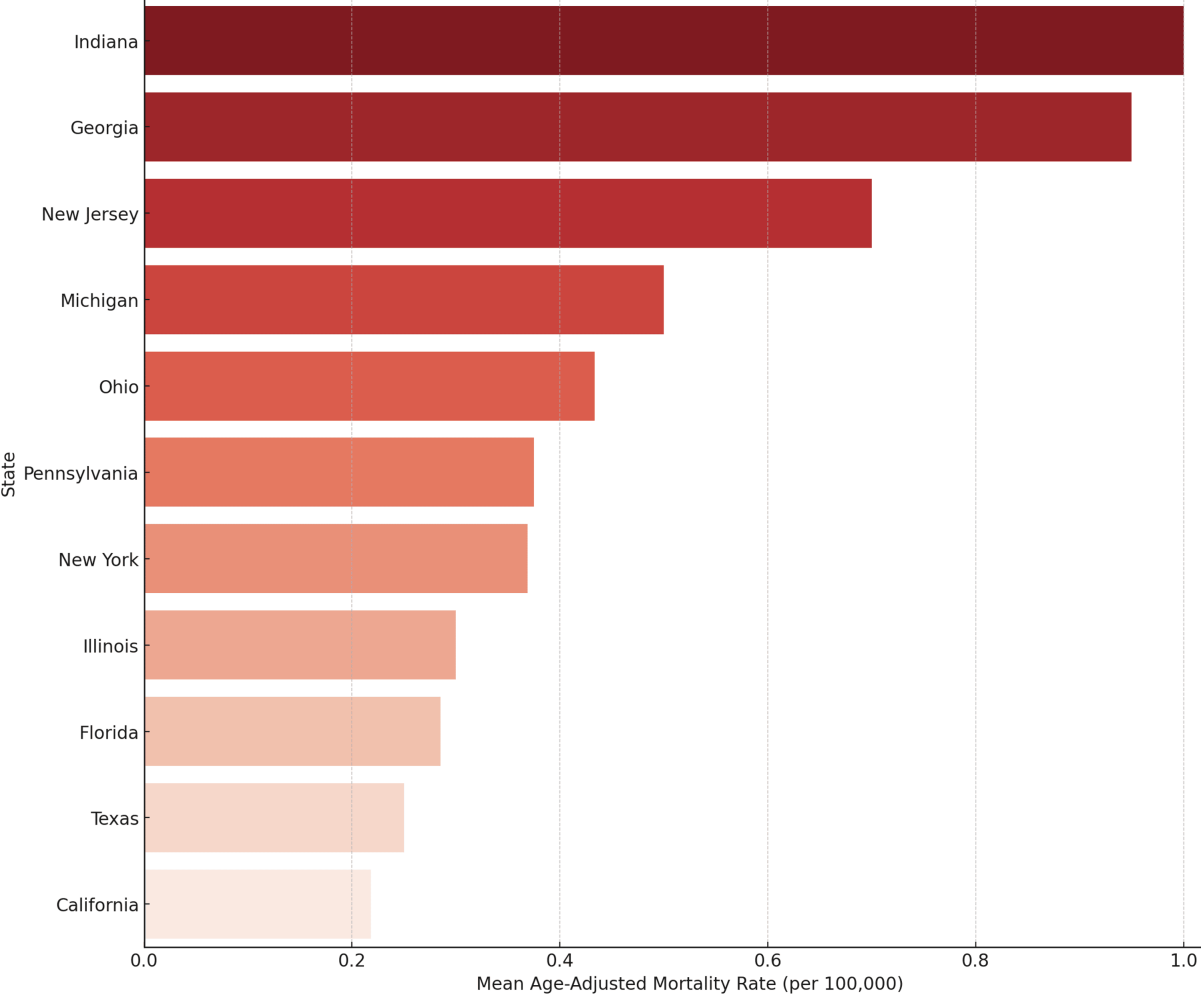
State-Level Mean Age-Adjusted Mortality Rate for Male Breast Cancer (1999-2020)

We figured out the mean age-adjusted death rates for each state. The bar chart in Figure 3 arranges the states in order of highest to lowest burden. States like Indiana, Georgia, and New Jersey had death rates that were much higher than the national average. These variations may be due to differences in how easy it is to get medical care, how often people get screened, or how late they are diagnosed.

Based on available data, only the White racial group had consistently reliable mortality estimates. The rate ratio for White males was set to 1.0 (reference), but analysis of other groups was limited due to small counts and suppression of unreliable rates. This finding underscores the need for improved data granularity in racial minority subgroups.

Multivariate regression

**Figure 4:**
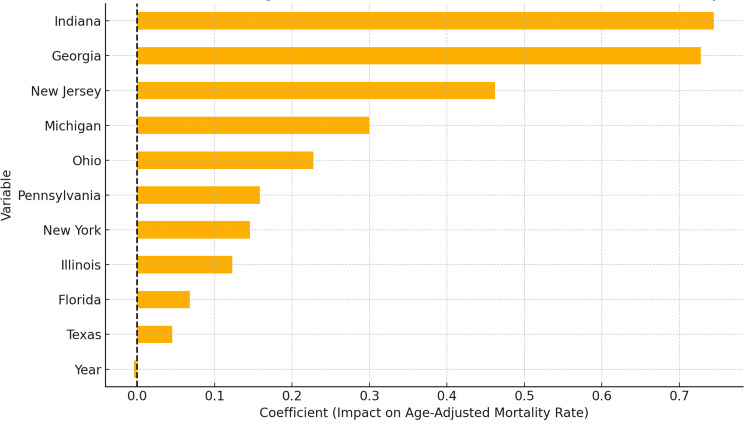
State-Level Effects and Temporal Trend (Year) on Age-Adjusted Mortality Rates, 1999-2020

The bar chart in Figure 4 shows how different factors, like specific states, affect the age-adjusted mortality rates in Indiana (β = 0.74) and Georgia (β = 0.73), highlighting states with much higher death rates. After adjusting for year and state, the model explained 88.9% of the variance in mortality (R² = 0.889, p < 0.0001).

## Discussion

MBC is rare but significant, and understanding its mortality trends and disparities is essential for improving healthcare delivery and outcomes. This analysis employs various methodologies to examine temporal and geographic patterns, primarily focusing on White males due to data limitations. Key findings are presented with visual evidence and comparisons to existing literature.

The overall decline in age-adjusted death rates aligns with improvements in early detection and systemic therapy, consistent with other studies. For example, Anderson et al. (2009) reported a 3.8% annual decrease in MBC mortality from 2000 to 2010, attributed to increased use of tamoxifen and advancements in adjuvant therapy [8]. Our significant annual percent change (APC) of -4.22% (1999-2007; p = 0.029) mirrors these findings. However, the sharp but non-significant decrease of -15.04% from 2008 to 2012 contrasts with European cohorts, where a steady decrease was observed [9]. This discrepancy may reflect differences in healthcare system integration or data quality. The plateau from 2013 to 2020 (APC = -1.79%, p = 0.46) contrasts with continuous declines in FBC, highlighting unique biological and therapeutic aspects of MBC. Wang et al. (2019) [10] proposed that current treatments might not work as well for rare tumors, and new trials focusing on BRCA2 mutations in MBC look promising but are not used enough, which could help explain the plateau [11]. Geographic disparities in mortality are evident, with Indiana, Georgia, and New Jersey reporting the highest rates (Figure 1). Conversely, states like California and Texas had lower rates (0.2-0.4 per 100,000), consistent with two other studies that found that centralized cancer care networks and Medicaid expansion led to better outcomes in these areas [12,13]. 

The sparse mortality data for non-White groups in our study reflects a systemic issue, well-documented in oncology research, highlighting that racial minorities are underrepresented in cancer registries due to underreporting and small sample sizes, limiting insights into disparities [14]. Our inability to analyze non-White groups prevents comparisons with studies like Yadukumar et al. (2023) [15], which found that Black males are often diagnosed at later stages, exacerbating treatment inequities. Efforts to improve racial/ethnic data collection may address this limitation in future research [16].

The omission of race and socioeconomic factors - common in registry-based studies - limits direct comparison with models that include social determinants, which is similar to two studies that linked mortality to income and education levels [17,18]. This limitation is consistent with Brawley (2002) [19], who emphasized the predictive value of socioeconomic factors in cancer outcomes. The reduction in mortality from 1999 to 2007 reflects the efficacy of hormone therapy, aligning with global trends [20].

The post-2013 plateau emphasizes the need to make male-specific treatment advancements, as noted in the ASCO guidelines [21]. Geographic disparities suggest the need for initiatives like the CDC’s National Breast and Cervical Cancer Early Detection Program, which has reduced inequities in female populations but remains underutilized for men. While MBC mortality has declined overall, progress remains uneven across time and geography.

Our results are in consonance with earlier research on treatment efficacy and geographic differences, but they also show unique plateaus linked to the extrapolation of FBC data [20,22]. To sustain mortality reductions, we recommend (1) addressing systemic inequalities by expanding Medicaid, (2) improving data infrastructure with mandatory registry reporting [23], and (3) developing androgen receptor-targeted therapies (42% actionable mutations [21]), all of which are outlined in the ASCO/SSO/SOBO guidelines in 2023.

Limitations

When looking at the outcomes of this study, it's important to keep in mind that it has some flaws. First, the CDC WONDER database is quite complete, but it only uses death certificate data, which may not always be accurate because of coding errors or underreporting, especially for less common diseases like MBC. Another problem with the dataset is that it doesn't include enough non-White racial groups. We mostly looked at White males, since we had excellent data on them. However, the smaller sample size for Black and other racial groups may have made our analyses less powerful for these groups, which makes it harder for us to draw conclusions about racial differences.

Although the retrospective nature of the study provides us with useful information about trends, it does not allow us to draw conclusions about their causes. It is also difficult to precisely link changes in death rates to actions or improvements in healthcare because there isn't enough clinical data (such as treatment options, stage at diagnosis, and comorbidities). Lastly, because MBC is so rare, the results may not apply to individual patients or smaller, regional populations. More research, using larger datasets and more targeted studies, is needed.

## Conclusions

In the U.S., MBC mortality experienced a slight initial decrease from 1999 to 2007, but it has since stabilized, highlighting significant geographic variations and a lack of race-specific statistics. States such as Indiana and Georgia exhibited significantly elevated death rates. The rarity of the disease constrains research and trial participation, and hence obstructs advancement. Considering these findings, three areas require urgent action. Clinicians must enhance attention and awareness to mitigate diagnostic delays in men, particularly those with genetic predispositions. Secondly, researchers should prioritize studies focused on males and guarantee the participation of men in breast cancer trials to produce evidence-based advice particular to this demographic. Third, public health organizations must execute focused educational and awareness initiatives for both healthcare practitioners and the public to eliminate the stigma surrounding MBC and encourage early detection.
